# Risk Factors for Colonization of *E. coli* in Atlantic Bottlenose Dolphins (*Tursiops truncatus*) in the Indian River Lagoon, Florida

**DOI:** 10.1155/2011/597073

**Published:** 2011-10-01

**Authors:** Adam M. Schaefer, Gregory D. Bossart, Marilyn Mazzoil, Patricia A. Fair, John S. Reif

**Affiliations:** ^1^Marine Mammal Research and Conservation Program, Harbor Branch Oceanographic Institute, Florida Atlantic University, Ft. Pierce, FL 34946, USA; ^2^Georgia Aquarium, Atlanta, GA 30313, USA; ^3^Center for Coastal Environmental Health and Biomolecular Research, NOS, NOAA, Charleston, SC 29142, USA; ^4^Department of Environmental and Radiological Health Sciences, College of Veterinary Medicine and Biomedical Sciences, Colorado State University, Fort Collins, CO 80532, USA

## Abstract

Opportunistic pathogens related to degradation in water quality are of concern to both wildlife and public health. The objective of this study was to identify spatial, temporal, and environmental risk factors for *E. coli* colonization among Atlantic bottlenose dolphins (*Tursiops truncatus*) inhabiting the Indian River Lagoon (IRL), FL between 2003 and 2007. Age, gender, capture location, coastal human population density, proximity of sewage treatment plants, number of septic tanks, cumulative precipitation 48 hrs and 30 days prior to capture, salinity, and water temperature were analyzed as potential risk factors. Highest *E. coli* colonization rates occurred in the northern segments of the IRL. The risk of *E. coli* colonization was the highest among the youngest individuals, in counties with the highest cumulative rainfall 48 hrs and in counties with the highest number of septic systems during the year of capture. The prevalence of colonization was the highest during 2004, a year during which multiple hurricanes hit the coast of Florida. Septic tanks, in combination with weather-related events suggest a possible pathway for introduction of fecal coliforms into estuarine ecosystems. The ability of *E. coli* and related bacteria to act as primary pathogens or cause opportunistic infections adds importance of these findings.

## 1. Introduction

The human population inhabiting the coastal areas in Florida increased by 75% from 1980 to 2003 and continues to expand [1]. This substantial growth creates environmental stress on adjacent marine ecosystems and is of particular concern along the Indian River Lagoon (IRL), which comprises 40% of Florida's east coast. Changes in the environment as a result of anthropogenic activities can influence the proliferation and distribution of fecal coliform bacteria and potential pathogens including *Escherichia coli *[2]. There is strong evidence to suggest a link between environmental stressors and infectious pathogens [3–6]. Specifically, increased burdens on sewage systems have the potential to increase the numbers of fecal coliform bacteria that ultimately reach coastal and estuarine waters. Fecal coliform bacteria are an important indicator system to assess the impact of anthropogenic pollution on coastal ecosystems and public health. 

Certain strains of *E. coli* are well-recognized threat to human health while others serve as an indicator of fecal pollution, particularly among individuals exposed to coastal waters through recreation or the consumption of contaminated seafood [7]. There are six major strains of *E. coli* with varying pathogenicity, production of enterotoxins, and plasmid virulence factors [8]. Transmission among humans occurs primarily through ingestion of contaminated food, water, or by contact with fomites. Infection with pathogenic strains of *E. coli* causes gastrointestinal disease which can include enteritis, hemorrhagic colitis, hemolytic uremic syndrome, and additional complications in compromised individuals [8]. Upon entering the environment, *E. coli *will persist, grow, and colonize both humans and wildlife. * E. coli* has been identified as the causative agent in multiple disease outbreaks associated with swimming and other recreational exposure to water in the United States and Canada [9–12]. 

Colonization with *E. coli *is not limited to human populations. The organism has commonly been cultured from a variety of aquatic wildlife including bottlenose dolphins, harbor seals, elephant seals, ducks, geese, and seagulls [13–16]. Previous research on *E. coli* among marine organisms has established that exposure to multiple environmental factors is associated with increased risk of colonization. Among filter-feeding shellfish (oysters, clams, and mussels), a strong seasonal trend in risk of colonization corresponding with increases in average water temperature and rainfall was identified [17, 18]. Similarly, the frequency of detection of fecal pathogens including *E. coli* increased during the rainy season among southern sea otters (*Enhydra lutris kenyoni*) [2].

Previous reports have identified *E. coli *colonization in bottlenose dolphins (*Tursiops truncatus) *from multiple geographic areas, including estuarine populations [15, 19, 20]. Colonization was reported to be as high as 69% of dolphins sampled in Charleston Harbor, SC and the Indian River Lagoon, FL (IRL) with 25% of the isolates resistant to one or more antibiotics [16]. However, no previous studies have attempted to determine whether the patterns of *E. coli* colonization among bottlenose dolphins are associated with environmental risk factors.

Therefore, the objective of this study was to identify environmental and other risk factors for *E. coli* colonization among free-ranging dolphins in the IRL. We incorporated the spatial distribution of members of this estuarine dolphin population with biological and environmental characteristics to explore exposures at an individual level. The large size of the estuary, varied land use bordering the lagoon, and availability of an extensive data base on the health of the bottlenose dolphin population, including microbiological data, makes this population well suited for investigation. To our knowledge, the current study is the first to examine the colonization of *E. coli* among dolphins using a spatial and temporal risk assessment model. 

## 2. Methods

### 2.1. Dolphin HERA Project

Data were collected as a part of the Bottlenose Dolphin Health and Risk Assessment Project (HERA), a collaborative multidisciplinary effort to assess health in two estuarine regions along the eastern coast of the United States [21]. All research was approved by the Harbor Branch Oceanographic Institution Animal Care and Use Committee. Dolphins were sampled from the IRL in June of each year between 2003 and 2007. Age was determined following extraction of a tooth under local anesthesia using previously described methods [22].

### 2.2. Sample Collection

Fecal samples for microbial isolation were collected using Amies culturette swabs (MML Diagnostics, Troutdale, OR). Fecal material was collected in sterile 15 mL conical vials opportunistically as the animal defecated and transferred to a sterile swab or by inserting the swab directly into the rectum. Swabs were stored on ice packs in coolers and shipped overnight to a commercial laboratory on cold packs.

### 2.3. Organism Identification

All microbial analyses were conducted by Micrim Laboratories, Fort Lauderdale, FL. Standard methods were used for organism identification from all fecal cultures, including growth on selective and differential media, colony morphology, gram stain, and biochemical reactions [23]. Additional microbial information generated from the HERA project has been described elsewhere [20]. *E. coli* was identified using standard methods including a gram stain and growth on Sorbitol-MacConkey agar and blood agar incubated for 24 hrs at 37°C [23]. Animals for which fecal samples showed heavy growth with *E. coli* as the dominant species were classified as colonized.

### 2.4. Antimicrobial Sensitivity Testing

Antibiotic resistance of *E. coli* was determined by disk diffusion assays [23]. The protocol included standardized inocula of bacteria swabbed onto the surface of Mueller-Hinton agar plates. Impregnated antimicrobial discs were placed onto the inoculated agar and the samples were incubated for 24 to 48 hours at 37°C. The tested agents were amikacin, amoxicillin-clavulanic acid, ampicillin, cefotaxime, ceftazidime, cephalothin, chloramphenicol, clindamycin, ciprofloxacin, enrofloxacin, erythromycin, furadantin, gentamicin, marbofloxacin, oxacillin, penicillin, piperacillin, septra/bactrim, and tetracycline. Zones of inhibition were measured, and the Clinical and Laboratory Standards Institute (CLSI) antimicrobial susceptibility testing standards M2-A9 and M7-A7 were applied.

### 2.5. Environmental Data

The IRL is a shallow-water ecosystem that encompasses 40 percent of Florida's east coast and is an aggregate of three estuaries. Due to limited tidal exchange, the lagoon is vulnerable to the influx of chemical and biological pollutants. Water quality in the IRL has deteriorated during the past five decades because of watershed alterations and changes in land drainage patterns. Decreased water quality is attributed primarily to fresh- and storm-water discharges that alter salinity and water clarity and introduce nutrients and pollutants into the system [24]. 

**Figure 1:**
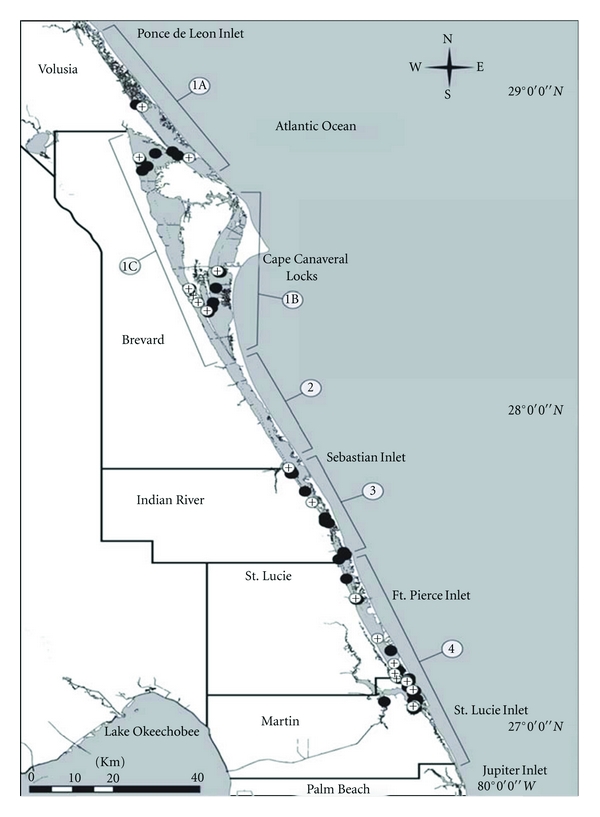
Spatial distribution of *E. coli *positive and negative dolphins with spatial segments by Florida counties in the Indian River Lagoon, FL.

The study area was divided into 6 segments based on hydrodynamic and geographic features (Figure 1) [25]. Dolphins were assigned to an IRL segment based on the individual's capture location during HERA. In addition, each dolphin was assigned to a county bordering the IRL based on the GPS coordinates of its capture location. Due to the high degree of site fidelity exhibited by the IRL dolphin population, capture location can be used to represent an approximation of each individual's relative distribution within the lagoon [26]. 

Human population density for each segment of the IRL was calculated using the 2000 US Census data for census tracts directly bordering the IRL [1]. Density was measured as inhabitants per square mile and categorized into four groups based on the data distribution. The number and location of sewage treatment facilities along the lagoon were calculated for each segment using data from the Florida Department of Environmental Protection online Geographical Information Systems (GIS) mapping resources [27]. Rainfall, water temperature, and salinity data were obtained from the water management districts' online databases [28, 29].

Additional environmental data were analyzed at the county level due to limited availability of local data for these parameters. Locations and totals of new and existing septic systems by year in which individuals were captured were obtained through the Florida Department of Health, Division of Environmental Health from county permit data. Forty-eight hour and 30-day cumulative rainfall were calculated using daily radar rainfall estimates (inches) from monitoring sites near the lagoon by county. Mean water temperature and salinity 30 days prior to capture were also calculated from monitoring stations located in the lagoon by county. Spatial mapping was done using ArcMap 9.3 (ESRI, Redlands, Calif, USA).

### 2.6. Statistical Analysis

Environmental risk factors included water temperature, salinity, population density, sewage treatment plants, septic systems, and rainfall. Dolphin age and human population density were categorized in quartiles based on the data distribution. Water temperature, salinity, population density, sewage treatment plants, septic systems, and rainfall were categorized into tertiles for analysis. 

Dolphins with *E. coli* colonization (cases) were compared to those from which *E. coli* was not cultured (controls). Logistic regression analyses were conducted to generate risk estimates for each quartile of exposure using the lowest quartile in each exposure category as the referent. Risk estimates were expressed as odds ratios (OR) with their 95% confidence intervals (CI). Univariate analysis was used to determine which variables were independent risk factors for colonization prior to inclusion in a multivariate model. Those independent environmental and demographic risk factors found to be statistically significant in the univariate analysis were included in the multivariate logistic model. A forward stepwise approach with the likelihood ratio test and a *P* value of <0.05 were used as inclusion criteria for covariates in the final model. Adjusted odds ratios and their 95% CI were generated for each independent variable. Model fit was assessed using the Hosmer and Lemeshow test [30]. Odds ratios with a 95% CI that did not include the null value of one were considered statistically significant at *P* < 0.05. All analyses were performed using PASW Statistics 17.0 for Windows (SPSS Inc., Chicago Ill, USA).

## 3. Results

A total of 96 individual dolphins, captured and released between 2003 and 2007 in the IRL, received fecal swabs and were included in the analysis. *E. coli* was isolated from 39 dolphins, (40.6%) 34 of which had the full suite of data necessary for this analysis. The observed prevalence of *E. coli* is identical to the 41% described by Buck et al. [19] using similar microbiology methods in a study of stranded cetaceans from the northeast and southeast US coasts. The animals included in this study are a subset of the complete HERA microbial screening of IRL and Charleston, SC dolphins reported previously [20]. Among the individual animal demographic variables examined for risk of colonization in univariate analysis, age contributed significantly with animals less than 6.5 years of age at the highest risk (OR = 3.8, 95% CI 1.5, 9.6) compared to individuals 16 years of age or older (Table 1). Gender was not associated with risk of colonization.

The prevalence of *E. coli *colonization differed significantly across the five years of dolphin sampling and was the highest in 2004 (62.1%) and the lowest in 2003 (8.3%). Animals cultured in 2004 and 2005 were 18.0 (95% CI 3.52, 91.9) and 7.7 (95% CI 1.35, 43.9) times more likely, respectively, to be positive for *E. coli* compared with those captured during 2003 (Table 2). 

The prevalence of colonization also differed spatially. Dolphins sampled from the northern segments of the IRL (1A, 1B, 1C) had a higher prevalence of *E. coli *in their feces (43.9%) than those sampled from the two southern segments (3 and 4) (29.6%), but the difference was not statistically significant. (*P* = 0.13 by Chi-squared analysis). However, a significant difference in the risk of colonization was found with dolphins sampled from the northernmost region of the lagoon (segment 1A) 4.3 (95% CI 0.97, 19.4) times more likely to have a positive culture compared to the southernmost segment (Segment 4). In contrast, two segments (3 and 1B) exhibited a reduced risk 0.29 (95% CI 0.0, 1.4) and 0.34 (95% CI 0.0, 1.7) when compared to the northernmost segment (1A) of the lagoon (Table 2, Figure 1). 

 There was little evidence that environmental factors were associated with the risk of *E. coli* colonization at the segment level (Table 3). Human population density and the number of sewage treatment plants in the segment did not contribute to the risk of colonization. Similarly, water temperature and salinity at the site of capture were not significant risk factors for colonization. However, several associations between environmental factors and risk of *E. coli* colonization in IRL dolphins were found in county-level analyses. The number of septic tanks in the county during the year of capture was significantly associated with *E. coli* colonization. Animals in areas with ≥82,244 tanks (mean 126,438) during the year of capture were 6.6 (95% CI 2.1, 20.5) times more likely to be colonized compared to those in areas with ≤70,286 septic tanks (mean 56,262). Precipitation of total 30 days prior to capture was not a significant risk factor. In contrast, animals captured when total rainfall 48 hr prior to capture was between 0.24 and 0.80 inches were 10.3 (95% CI 2.9, 36.9) times more likely to be colonized compared to captures with rainfall ≤0.23 and ≤0.81 inches. However, the risk of colonization was not significantly increased for the highest level of 48 hr precipitation >0.81 inches.

In the final multivariate model age, the number of septic tanks and 48 hr precipitation were included as risk factors. Septic tanks were significantly associated with colonization when adjusted for the remaining covariates with a stepwise increase in risk to 6.3 (95% CI 1.0, 39.7) amongst the highest totals. The highest two categories of total 48 hr precipitation both contributed to an increased risk of colonization (OR 8.4, 95% CI 2.0, 35.4 and OR 9.0, 95% CI 1.2, 70.2) (Table 4). Animals 6.5 years or younger were 8.6 times more likely to be colonized (OR 8.6, 95% CI 1.3, 57.3) compared to older individuals after adjusting for other covariates.

## 4. Discussion

*E. coli* is a ubiquitous organism and has been isolated from feces obtained from bottlenose dolphins in multiple geographic areas [16, 19, 20]. The overall prevalence of *E. coli* in IRL dolphins between 2003 and 2007 was 52%, which is similar to the previously reported prevalence of 48% from the IRL in 2003 [16].

The finding that prevalence of *E. coli* colonization was the highest among the youngest age group of dolphins is consistent with the development of acquired immunity against this bacterium. The immune response to gastrointestinal infection with *E. coli* is mediated primarily through IgA secreted by the gut-associated lymphoid tissue located within the small intestine [13]. Repeated exposures to the organism during the first years of life appear to contribute to the development of acquired immunity over time as shown in humans in developing countries where childhood infection with enterotoxigenic strains is a major public health problem [31]. 

The spatial differences in colonization are of interest because the northern portion of the lagoon is less urbanized relative to the southern regions. Water temperature and salinity showed little variability between IRL segments and was not associated with colonization. This is in contrast to a previous report on risk factors for human enteric infections which demonstrated an increase in infections associated with higher ambient temperatures [32].

Increased precipitation associated with increased microbial colonization has been reported for some coastal species including shellfish and southern sea otters (*Enhydra lutris nereis) *[2, 17, 18]. We found an association between colonization of *E. coli* and rainfall during the 48 hrs prior to sampling but not with precipitation 30 days prior to sampling suggesting that runoff from terrestrial sources of fecal contamination from wildlife, domestic animals, or humans could contribute to the risk of enteric infection in estuarine dolphins. 

Among the anthropogenic factors examined, human population density was not associated with an increase risk of colonization. Similarly, the risk of colonization with antibiotic-resistant *E. coli* in stranded northern elephant seals along the California coastline did not follow a dose-response pattern with human population density [33]. This is in contrast to a previous study that determined an increased risk of human illness associated with increased exposure to coastal waters near densely populated areas compared to less developed coastlines [34]. 

The number of septic tanks in the county of capture was a strong risk factor for colonization, with odds ratios over 6 for the highest category in both univariate and adjusted analyses. Further, the northern regions of the lagoon where *E. coli* colonization was most prevalent also had a larger number of septic tanks compared to the southern portions. When compared to areas on sewer systems, high concentrations of septic tanks show elevated levels of seepage [35]. Increased septic tank usage has been associated with higher fecal coliform bacteria densities in other coastal environments [36, 37].

Animals sampled during 2004 and 2005 were more likely to be colonized compared to 2003 and 2006-2007. The peak in colonization coincided with multiple hurricanes and water discharge events from inland lakes along the Florida coast in 2004. The resulting rain, discharge, wind, and erosive wave action likely created ideal conditions for washing of feces-contaminated water into the lagoon from low-lying septic systems and possible release of bacteria from the sediment. Previous studies have identified increases in fecal coliform counts as a result of hurricane events in the United States [38, 39]. An increase in fecal coliforms was also documented in areas without post hurricane flooding [40]. Non point sources, specifically land runoff have been associated with septic seepage [41]. Septic seepage associated with hurricane events may have been responsible for the spatial and temporal increase in *E. coli *colonization observed in the study population.

The current study is limited by the cross-sectional design which did not permit longitudinal assessment of *E. coli *colonization to determine whether these were transient or more permanent events. Seasonal changes in bacterial counts and rainfall were not reflected because all dolphin sampling took place during June and July. Finally, this study was unable to distinguish commensal infections with *E. coli* from those that may have been pathogenic. 

 The study of fecal coliform bacteria in estuarine bottlenose dolphins is a useful barometer of ecosystem health and anthropogenic impacts on the local environment. Our results suggest that anthropogenic influences exacerbated by meteorological events are significant risks factors associated with *E. coli* colonization among bottlenose dolphins. Bottlenose dolphins are an important sentinel species for humans since these estuarine animals are exposed to many of the same environmental hazards as people who live in coastal areas or use the marine environment recreationally [42]. Therefore, the environmental risk factors identified for *E. coli* colonization in this dolphin population may represent shared risk that has impact on public health.

## Figures and Tables

**Table 1:** Univariate analysis of individual risk factors for positive and negative *E. coli* colonization among bottlenose dolphins (*n* = number of individual dolphins).

	*E. coli* + *n* (%)	*E. coli* − *n* (%)	OR (95% CI)
Age*			
<6.5	11 (50.0)	11 (50.0)	3.8 (1.5, 9.6)
6.5–10.9	11 (39.3)	17 (60.7)	1.9 (0.7, 4.8)
11.0–15.9	9 (39.1)	14 (60.9)	2.5 (1.0, 6.6)
>16.0	3 (18.7)	13 (81.3)	1.00

Gender			
Female	10 (35.7)	18 (64.3)	1.00
Male	24 (35.3)	44 (64.7)	1.2 (0.6, 2.3)

*Ages available for 55 of 62 *E. coli.*

**Table 2:** Distribution of *E. coli *positive and negative dolphins in the Indian River Lagoon, FL, by segment and year of capture with odds ratios and 95% confidence intervals.

	*E. coli *+ *n* (%)	*E. coli* − *n* (%)	OR (95% CI)
Segment			
4	14 (35.0)	26 (65.0)	1.00
3	2 (13.3)	13 (86.7)	0.3 (0.1, 1.4)
1C	9 (50.0)	9 (50.0)	1.8 (0.6, 5.7)
1B	2 (15.4)	11 (84.6)	0.3 (0.1, 1.7)
1A	7 (70.0)	3 (30.0)	4.3 (1.0, 19.4)

Year of capture			
2003	2 (8.3)	22 (91.7)	1.00
2004	18 (62.1)	11 (37.9)	18.0 (3.5, 91.9)
2005	7 (41.2)	10 (58.8)	7.7 (1.3, 43.9)
2006	5 (29.4)	12 (70.6)	4.6 (0.8, 27.3)
2007	2 (22.2)	7 (77.8)	3.1 (0.4, 26.6)

**Table 3:** Univariate analysis of environmental risk factors for *E. coli* colonization among bottlenose dolphins.

	*E. coli* + *n* (%)	*E. coli* − *n* (%)	OR (95% CI)
Segment level analysis			
Sewage treatment facilities			
≤8	9 (26.5)	14 (22.6)	1.00
9–19	11 (32.4)	22 (35.5)	0.8 (0.3, 2.3)
≥20	14 (41.2)	26 (41.9)	0.8 (0.3, 2.4)

Population density			
<595.39	9 (26.5)	14 (22.6)	1.00
595.39–754.39	9 (26.5)	9 (14.5)	1.6 (0.4, 5.4)
754.40–771.41	2 (5.9)	13 (21.0)	0.2 (0.0, 1.3)
≥771.42	14 (41.2)	26 (41.9)	0.8 (0.3, 2.4)

County-level analysis			
48 hr precipitation (*inches*)*			
≤0.23	3 (13.3)	24 (46.4)	1.00
0.24–0.80	19 (63.3)	12 (21.4)	10.3 (2.9, 36.9)
≥0.81	7 (23.3)	18 (32.1)	2.5 (0.6, 9.9)

30 day precipitation (*inches*)			
≤5.72	16 (47.1)	20 (32.3)	1.00
5.73–6.24	4 (11.8)	26 (41.9)	0.2 (0.1, 0.7)
≥6.25	14 (41.2)	16 (25.8)	1.1 (0.4, 2.9)

Water temperature (°C)*			
≥30.2	12 (48.0)	21(39.6)	1.00
29.4–30.1	12 (48.0)	15 (28.3)	1.4 (0.5, 4.0)
28.4–29.39	1 (4.0)	17 (32.1)	0.1 (0.0, 0.9)

Salinity (ppt)*			
≤23.01	11 (44.0)	20 (37.7)	1.00
23.02–22.84	3 (12.0)	26 (49.1)	0.2 (0.1, 0.8)
≥33.85	11 (44.0)	7 (13.2)	2.9 (0.7, 9.5)

Septic tanks per year			
≤70.286	7 (20.6)	27 (43.5)	1.00
70.287–82.243	10 (29.4)	25 (40.3)	1.5 (0.5, 4.7)
≥82.244	17 (50.0)	10 (16.1)	6.6 (2.1, 20.5)

*Data not available for all individuals.

**Table 4:** Multivariate logistic regression analysis of risk factors for *E. coli* colonization among bottlenose dolphins.

	Adjusted OR	95% CI	*P* value
Age			
>16.0	1.0	—	—
11.0–15.9	3.5	0.6, 19.6	0.15
6.5–10.9	5.3	0.9, 30.1	0.06
<6.5	8.6	1.3, 57.3	0.03

48 hr precipitation (*inches*)**			
≤0.23	1.0	—	—
0.24–0.80	2.1	2.0, 35.4	< 0.01
≥0.81	2.2	1.2, 70.2	0.04

Septic tanks per year**			
≤70.286	1.0	—	—
70.287–82.243	1.9	0.2, 15.4	0.53
≥82.244	6.3	1.0, 39.7	0.05
